# Ultrasensitive Time-Resolved Fluoroimmunoassay for Saikosaponin a in Chaihu (Bupleuri Radix)

**DOI:** 10.1371/journal.pone.0151032

**Published:** 2016-03-11

**Authors:** Zhi Chao, Qian Cui, Enwei Tian, Weiping Zeng, Xuan Cai, Xiaolei Li, Hiroyuki Tanaka, Yukihiro Shoyama, Yingsong Wu

**Affiliations:** 1 School of Traditional Chinese Medicine, Southern Medical University, Guangzhou 510515, China; 2 Graduate School of Pharmaceutical Sciences, Kyushu University, Fukuoka, Japan; 3 Faculty of Pharmaceutical Sciences, Nagasaki International University, Sasebo 859–3298, Japan; 4 School of Biotechnology, Southern Medical University, Guangzhou 510515, China; Jadavpur University, INDIA

## Abstract

The aim of this study is to establish a time-resolved fluoroimmunoassay (TRFIA) system for quantitative analysis of saikosaponin a (SSa) in the crude drug of Chaihu (Bupleuri Radix). A 96-well microplate coated with rabbit anti-mouse IgG was incubated with the methanol extracts of Chaihu samples and a mouse anti-SSa monoclonal antibody, and a Eu^3+^-labeled SSa-human serum albumin conjugate was used as the tracer. The established competitive TRFIA showed a good fourth order polynomial fitting from 0.01 to 10.0 *μ*g/mL for standard SSa sample with a detection limit of 0.006 *μ*g/mL. The intra- and inter-assay coefficients of variation of the assay were 7.3% and 8.9%, respectively, and the average SSa recovery was 119.2%. For samples of Chaihu extract, the results of this assay showed a good correlation with those by enzyme-linked immunosorbent assay established previously. This TRFIA system is ultrasensitive for detecting SSa with a wide detection range and a good stability and represents the first attempt of using TRFIA for quality evaluation of the crude drug of Chaihu.

## Introduction

**Fig 1:**
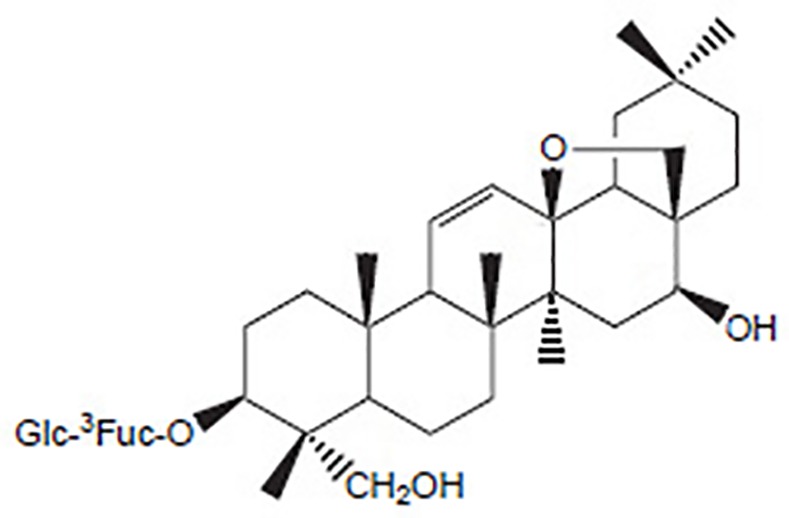
Structure of saikosaponin a.

Chaihu (Bupleuri Radix), a common traditional Chinese medicinal herb derived from the dried roots of *Bupleurum chinense* DC. or *B*. *scorzonerifolium* Willd., has been used for medical purposes in China for more than 2000 years. According to ancient Chinese medical literatures, Chaihu is capable of regulating the exterior and interior metabolisms, dispersing evil heat from the superficies, soothing the liver, and promoting *yang* and *qi* (representing “life energy” or “life force” in Traditional Chinese Medicine theories) [1, 2]. Among the complex constituents, saikosaponins, which have a typical oleanan-type skeleton as the aglycon, have been identified by modern techniques as the major biological active constituents in Chihu. Of these saikosaponins, saikosaponin a (SSa) (Fig 1), a major saponin, has been shown to possess versatile bioactivities to suppress inflammation [3] and oxidation, protect liver function [4], induce tumor cell apoptosis, inhibit carcinogenesis [5–9], and induce cell differentiation [10]; research evidence also demonstrated its activities in immunomodulation [11], promoting corticosterone secretion [12], and lowering plasma cholesterol [13]. Because of the uneven quality of Chaihu in the market [2], quantification of SSa is critical to ensure the effectiveness of the crude drug [1], thus an accurate, sensitive, and convenient method for determination of SSa in Chaihu is essential.

Several techniques have been developed for analyzing SSa in Chaihu and various Chaihu products, including thin-layer chromatograph scanning (TLCS) [14], high-performance liquid chromatography (HPLC) [15–19], HPLC coupled with evaporative light scattering detector (ELSD) [20], HPLC coupled with mass spectrometry [21], ultraperformance liquid chromatography (UPLC) [22], UPLC coupled with mass spectrometry [23, 24], and capillary electrochromatography [25]. Chromatography-based analytical techniques are the most frequently used modality for quantitative and/or qualitative analysis of SSa. But because SSa has a rather short maximum absorption wavelength (205 nm), interference easily occurs in SSa detection using ultraviolet or diode array detector (DAD) to lower the detection sensitivity. In addition, this strategy also requires sophisticated equipment (eg, a mass spectrometer), complicated sample pretreatment, and the use of toxic organic solvents in the mobile phase.

Immunological approaches provide valuable alternatives for SSa analysis. The first attempt of immunologically based SSa detection was made by Jung *et al*., who used anti-SSa polyclonal antibody for quantitative analysis of SSa [26]. But as polyclonal antibodies recognize non-specific antigenic determinants and also cross-react with the non-target proteins, the specificity of the assay was considerably lowered. In a more recent study, an enzyme-linked immunosorbent assay (ELISA) system was established using an anti-SSa monoclonal antibody (MAb) [27]. Due to the high specificity of the monoclonal antibody, this assay was successfully applied in quality inspection of commercial Chaihu crude drugs [28].

Different from the approaches described above, time-resolved fluoroimmunoassay (TRFIA) represent an ultrasensitive technique using lanthanide elements and their chelates as the tracer with unique fluorescence properties. This technique is commonly used in laboratory medicine, agriculture, food industry, environmental health study, and forensic science [29–32]. But currently few studies have been reported to describe its application in crude drug analysis. In this study, we aimed to establish a TRFIA-based system for SSa detection for providing an accurate and more sensitive, less time-consuming method for quality control of Chaihu.

## Materials and Methods

### Chemicals

Standard SSa sample was purchased from National Institutes for Food and Drug Control, China. Bovine serum albumin (BSA) and human serum albumin (HSA) was obtained from Sigma-Aldrich (St. Louis, USA). Rabbit anti-mouse IgG was purchased from Rockland Immunochemicals (Limerick, USA). A previously prepared murine monoclonal antibody 1G6 against SSa was used [27], which was a highly specific IgG with a light chain of κ-type and showed low cross-reactions with saikosaponin c, d and b2 (at rates of 2.65%, 3.76%, and <0.25%, respectively). The crude drug samples of Chaihu were purchased from the local pharmacies. Based on the macroscopic characteristics and the description in Chinese Pharmacopoeia, the samples were identified by the author (ZC) as the roots of *B*. *chinense*. Samples No.4 and No.5 were of poor quality because of the presence of the stem remnants to suggest apparent adulteration. The other chemicals used were routine reagents of the analytical grade.

### Synthesis of Antigen-Carrier Protein Conjugate

SSa-HSA conjugate was synthesized using a model based on the periodate oxidation method. Briefly, the standard sample of SSa (3.2 mg) was dissolved in 0.5 mL of methanol (MeOH), in which a sodium periodate (10.0 mg)/H_2_O (1.0 mL) solution was added dropwise. The mixture was stirred at room temperature for 1 h to allow the oxidative reaction, followed by the addition of HSA (5.0 mg) in 50 mM carbonate buffer (1.0 mL; pH 9.6) and further stirring at room temperature for 12 h. The reaction mixture was dialyzed against H_2_O at 4°C for 2 days and then lyophilized to obtain SSa-HSA conjugate.

### Eu^3+^ Labeling of Antigen-Carrier Protein Conjugate

The synthesized SSa-HSA conjugate was dissolved in the labeling buffer containing 50 mM Na_2_CO_3_-NaHCO_3_ (pH 8.5) and 155 mM NaCl and gently mixed with Eu^3+^-DTTA (isothiocyannatobenzyl diethylenetriaminetetraacetic acid) dissolved in the same buffer. The mixture was vortexed gently at room temperature for 16–20 h before being loaded onto a sepharose CL-6B column (Pharmacia Company, Chalfont St Giles, UK) for purification. The column was eluted with the elution buffer (containing 50mM Tris-HCl, pH7.8, 0.9% NaCl and 0.05% NaN_3_), and the fractions from the first peak with the highest Eu^3+^ count were pooled. BSA solution was then added at a final concentration of 0.2% into the pooled fractions. Finally, the Eu^3+^-labeled SSa-HSA conjugate was aliquoted and stored at 4°C until use.

### Competitive TRFIA

**Fig 2:**
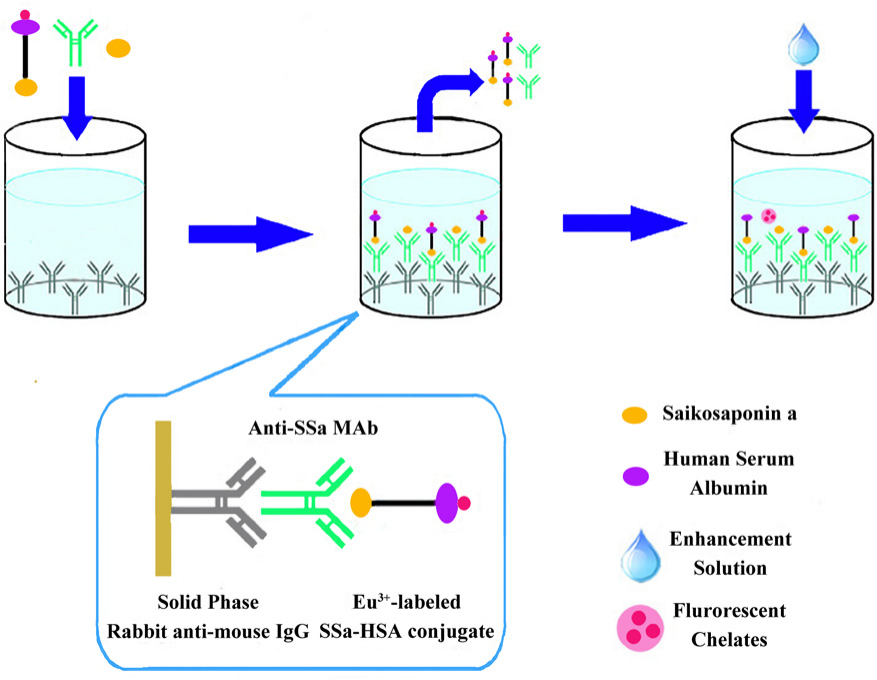
Schematic diagram of the TRFIA system for SSa detection. Rabbit anti-mouse IgG (“Y” shape in grey) was coated onto the plate; SSa (yellow oval dot), anti-SSa MAb (“Y” shape in green), and Eu^3+^ (red dot) -labeled SSa-HSA (purple oval dot) conjugate were mixed and incubated; after washing off of the extra reactants, the enhancement solution (blue drip) was added and the fluorescence intensity was measured.

Fig 2 shows the schematic diagram of the TRFIA system for SSa detection. The 96-well immunoplate (Nunc, Roskilde, Denmark) was coated with 100 *μ*L of rabbit anti-mouse IgG (4 *μ*g/mL) dissolved in 50 mM carbonate buffer (pH 9.6) at 4°C overnight, and then blocked with 300 *μ*L of the blocking buffer (50 mM Tris-HCl, 0.9% NaCl, 0.1% BSA and 0.05% NaN_3_, pH 7.8) to reduce the nonspecific reactions. In each well, 25 *μ*L of standard SSa samples at different concentrations or test samples dissolved in 10% MeOH solution were added and incubated for 1 h with 100 *μ*L of anti-SSa MAb solution (1:4000) and 100 *μ*L of Eu^3+^-labeled SSa-HSA conjugate (1:800). After three washings with the washing buffer (50 mM Tris-HCl, 0.9% NaCl, 0.1% BSA, 0.05% Tween 20 and 0.05% NaN_3_, pH 7.8), 200 *μ*L of the enhancement solution (100 mM acetate-phthalate buffer, pH 3.2, containing 0.1% Triton X-100, 15 *μ*M β-naphthoyltrifluoroacetate, and 50 *μ*M tri-*n*-octylphosphine oxide) was added to each well and incubated for 5 min. The fluorescence intensity was measured on a Victor^3^ 1420 Multilabel Counter (PerkinElmer, Waltham, USA) equipped with filters for Eu^3+^ (613 nm). The excitation wavelength was 340 nm and the emission wavelength was 613 nm with a delay time of 0.40 ms, window time of 0.40 ms, and cycling time of 1.0 ms. Curve fitting and calculation of the concentrations in the samples were performed using Multicalc software (PerkinElmer, Waltham, USA), where a spline algorithm on logarithmically transformed data was employed.

### Preparation of Test Samples and Standard Solutions

All the commercial Chaihu samples were prepared into dried powder using a steel grinder. The dried Chaihu powder (20 mg) was extracted with 0.5 mL of MeOH containing 5% NH_4_OH with sonication for 10 min, and the extract was centrifuged at 9000 *g* for 5 min to collect the supernatant. For each sample, the extraction was repeated six times. The pooled supernatants from six rounds of extraction were evaporated with N_2_ gas, and the residue was dissolved in 5.0 mL MeOH and diluted at 1:10 with H_2_O to obtain the test sample solution.

The standard SSa sample was precisely weighed and dissolved in MeOH to prepare a 5.0 mg/mL stock solution. Gradient concentrations of the stock solution were prepared by serial dilutions with 10% MeOH (0.001, 0.002, 0.005, 0.01, 0.02, 0.05, 0.10, 0.20, 0.40, 0.80, 2.0, 5.0, 10.0 and 20.0 *μ*g/mL) as the SSa standards. The test sample and standard SSa solutions were used for subsequent TRFIA and ELISA analysis.

### Calibration Curve

Serially diluted SSa standard solutions at 14 different concentrations (0.001 to 20.0 *μ*g/mL) were used to establish the calibration curve. The detection limit was determined by calculating the minimum detectable amount of standard SSa that could be significantly distinguished from zero (mean binding at zero dose at two times of the SD) [33, 34].

### Intra- and Inter-assay Variation

The intra-assay variation was tested by determining the percent coefficient of variations (%CVs) for 3 SSa samples of varying concentrations in 10 duplicate wells across the microtiter plate. The inter-assay variation was determined by evaluating the SSa samples in triplicate plates on 3 consecutive days using the same reagent lots, and the %CVs were calculated.

### Recovery Experiments

Various amounts of the standard SSa sample (25, 50, and 100 *μ*g) were spiked into dried Chaihu crude drug powder (20 mg), and the total SSa was extracted for detection following the procedures described previously. For each level, three replicates were analyzed.

### Correlation between TRFIA and ELISA Analysis

ELISA of the samples following the previously reported protocols was conducted to validate the results of the established TRFIA [27, 28]. In brief, a 96-well immunoplate was coated with 100 *μ*L of 1 *μ*g/mL SSa-HSA and blocked for 1 h with 300 *μ*L of phosphate buffered saline (PBS) containing 5% skim milk. Fifty microliters of various concentrations of the standard SSa sample or the test samples dissolved in 10% MeOH solution were incubated with 50 *μ*L of anti-SSa MAb solution (1:1000) for 1 h. The plate was washed 3 times with PBS containing 0.05% Tween 20, and then 100 *μ*L of 1: 1000 diluted peroxidase-labeled anti-mouse IgG was added and incubated for 1 h. Finally, 100 *μ*L of the substrate solution [0.1 M citrate buffer (pH 4.0) containing 0.003% H_2_O_2_ and 0.3 mg/mL 2,2’-azino-bis(3-ethylbenzothiazoline-6-sulfonic acid) diammonium salt (ABTS)] was added to each well and incubated for 10 min. The absorbance was measured by a microplate reader at 405 nm. The interpolated concentrations of the samples obtained by each method were then compared by correlation analysis.

### Data Analysis

The data were analyzed using the Statistical Package for Social Sciences (SPSS, version 19.0). For correlation studies, Spearman correlation coefficient was employed and the level of significance was set to alpha = 0.01. Paired signed rank test was used to determine the statistical significance between the results by TRFIA and ELISA (alpha = 0.05).

## Results

### Assay Sensitivity

**Fig 3:**
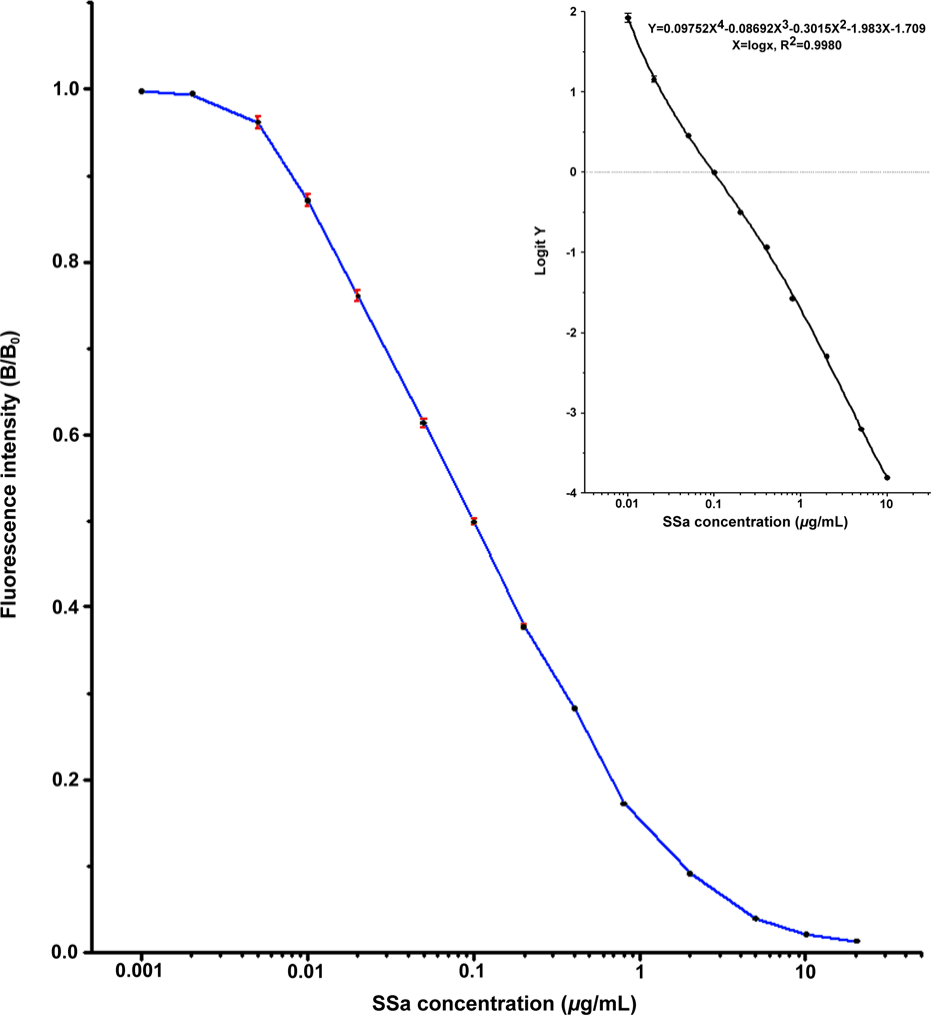
Standard curve of SSa content detected by TRFIA. The main body represents the dose-response curve between SSa concentrations and the reporting fluorescence values (B/B_0_), whereas the inserted smaller figure demonstrates the calibration curve acquired after log-logit treatment, showing a good fourth order polynomial fit. Each point represents the mean±SD of 10 assays in duplicate.

The dilution of Eu^3+^-labeled SSa-HSA conjugate that emitted 75% of the full fluorescence intensity (saturate solid phase fluorescence) was chosen as the working titer. A dose-response curve was obtained by averaging 10 individual curves normalized by the reporting fluorescence values (given as %B/B_0_, where B was the mean fluorescence intensity for each standard and B_0_ the mean fluorescence intensity for a zero SSa concentration). The calibration curve (Fig 3, S1 File) derived by processing the data of SSa concentration and the fluorescence intensity with log-logit function derived from fourth order polynomial fitting (logit *Y* = ln [*Y*/(1-*Y*)], *Y* = B/B_0_) [34] showed a detection range of 0.01–10.0 *μ*g/mL for SSa detection. The detection limit calculated from 3 curves, each prepared in duplicate, was 0.006 *μ*g/mL. Compared with the conventional ELISA, which had a detection range of SSa from 0.026 to 1.5 *μ*g/mL [27] (0.16–2.5 *μ*g/ml in another case [28]), the TRFIA system showed a sensitivity about 3–15 times greater and a much wider detection range.

### Assay Variation

The intra-assay (well-to-well) and inter-assay (plate-to-plate) variations were determined to assess the reproducibility and precision of this assay. The intra- and inter-assay maximum relative standard deviations (RSDs) were 7.9% and 11%, respectively (Table 1).

**Table 1:** Variations among TRFIA Runs for Analysis of SSa.

SSa (*μ*g/mL)	CV (%)	
	intra-assay (n = 10)	inter-assay (n = 3)
0.02	7.9 ± 0.02	8.5
0.20	6.3 ± 0.20	7.3
2.00	7.7 ± 3.9	11

### Recovery of SSa by Competitive TRFIA in Spiked Samples

Chaihu crude drug sample No.2 was randomly selected for the recovery experiment. Various amounts of standard SSa sample (25, 50, 100 *μ*g) were added to 20.0 mg dried crude drug powder (containing 116.9 *μ*g SSa after calculation), and the total amount of SSa was measured by competitive TRFIA. The recovery of spiked SSa was calculated as follows:

Recovery=(measured amount of SSa−116.9)/spiked amount×100%

The recovery rates were between 108.9% and 126.9% with reasonable RSD values (9.8% − 14%), as shown in Table 2. The average recovery rate was 119.2%.

**Table 2:** Recovery of SSa Determined by TRFIA in Spiked Samples.

spiked level (*μ*g)	measured amount ^a^ (*μ*g)	recovery ^a^ (%)	RSD (%)
25	144.1 ± 2.7	108.9 ± 11	10
50	177.9 ± 6.0	122.0 ± 12	9.8
100	243.8 ± 18	126.9 ± 18	14

^*a*^ Data are mean ± SD from triplicate analyses for each sample.

### SSa Contents in Chaihu Crude Drug Samples Determined by TRFIA

Table 3 shows the results of quantitative analysis of SSa contents in the crude drug samples of Chaihu purchased from the local pharmacies. In the 10 commercial Chaihu samples, the measured SSa contents in the methanol extract ranged from 0.27 to 8.77 *μ*g/mg, and 2 samples showed SSa contents lower than the minimum requirement documented in the Chinese Pharmacopeia.

**Table 3:** SSa contents in Chaihu samples determined by TRFIA and ELISA.

Sample no.	SSa contents determined by TRFIA (*μ*g/mg)	SSa contents determined by ELISA (*μ*g/mg)
1	8.74 ± 0.74	6.98 ± 0.33
2	5.85 ± 0.50	5.62 ± 0.11
3	8.69 ± 1.40	7.19 ± 0.08
4	1.10 ± 0.42	1.16 ± 0.86
5	0.27 ± 0.01	0.37 ± 0.28
6	7.17 ± 0.25	5.51 ± 0.16
7	2.89 ± 0.23	2.78 ± 0.23
8	4.72 ± 0.40	5.75 ± 0.42
9	5.49 ± 0.15	4.75 ± 0.17
10	8.77 ± 0.47	8.88 ± 0.66

Data are mean ± SD from triplicate analyses for each sample

### Correlation between TRFIA and ELISA for Detecting SSa Contents in Chaihu Crude Drug Samples

**Fig 4:**
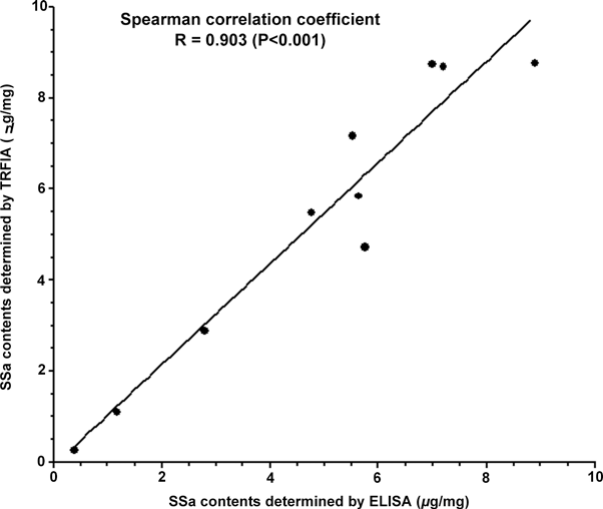
Correlation of SSa contents in Chaihu crude drug samples determined by TEFIA and ELISA. SSa contents in Chaihu crude drug samples determined by TRFIA (*Y*-axis) were well correlated with those determined by ELISA (*X*-axis), with a Spearman correlation coefficient (*R*) of 0.903 (*P* < 0.01).

For crude drug samples, SSa contents determined by TRFIA were similar to those by ELISA (Table 3, S1 File). The Spearman correlation coefficient (*R*) between the results by TRFIA and ELISA was 0.903 (*P*<0.01, Fig 4, S1 File). Paired signed rank test showed no significant difference between the SSa contents measured by the two methods (*P* = 0.153).

## Discussion

Chromatography-based approaches represent the current mainstream for saikosaponin analysis. As the maximum absorbance wavelength of SSa is 205 nm (near the end of the ultraviolet spectrum), ultraviolet- or DAD-based detection of SSa has a low sensitivity and requires a high purity of the mobile phase, complicated sample pretreatment, and sophisticated equipment such as an evaporative light-scattering detector and even a mass spectrometer. The immunological approaches are free of such limitations of chromatographical methods and allows more convenient assay of biomedical samples. In fact, the previously established ELISA has been shown to allow simultaneous analysis of SSa for a large number of samples [27, 28].

The TRFIA system using anti-SSa MAb we established showed a wide detection range (0.01–10.0 *μ*g/mL) and a low detection limit (0.006 *μ*g/mL), and was about 3–15 times more sensitive than ELISA. This assay was also less time-consuming than ELISA (by at least 1 hour) because of the omission of a secondary antibody. The validation experiments confirmed the sensitivity, reproducibility, and accuracy of the TRFIA system. The maximum intra- and inter-assay variations of this system (7.9% and 11%, respectively) were both <15%, suggesting its sufficient precision and good repeatability for SSa detection [35]. The recovery rates (between 108.9% and 126.9%, 119.2% on average) met the acceptance criteria (80%– 120%), and thus confirmed the accuracy of this assay system [36, 37].

Nevertheless, we noted a higher level of imprecision (higher %CV) of this new assay at higher SSa concentration levels compared with ELISA. Because of the greater sensitivity of the established TRFIA, even small variations of SSa content in the test samples would be greatly amplified, which may lead to a high %CV in detection. We adopted a simplified calibration curve with 5 standards to allow a high-throughput detection of SSa in samples; this, however, may give rise to the limitation of the assay precision and lead to a larger CV. Moreover, the skills in sample manipulation such as pipetting also affect the variation margin, especially for high SSa content samples.

We tested SSa contents in 10 commercial Chaihu samples using the established TRFIA system. The results were similar to those reported previously [28], which confirmed the validity and reliability of the TRFIA system. Samples No. 4 and No. 5 were found to have low SSa contents (below the minimal limit prescribed in Chinese Pharmacopoeia) because of serious adulteration with the aerial parts of the plant that contained no saikosaponins. This result is consistent with our macroscopic inspection of the crude drug before TRFIA determination.

Correlation between TRFIA and ELISA for detecting SSa contents in Chaihu crude drug samples was investigated. Commonly, linear regression is adopted in calibrating the concentration and absorbance in ELISA, but in this case, the data collected for ELISA showed a non-linear curve. We found fifth order polynomial fit model could provide a better fitting.

The correlation coefficient between the results of TRFIA and ELISA was 0.903 (*P*<0.01), demonstrating a high correlation between them and hence the good reliability of TRFIA. Paired signed rank test also confirmed the comparability between the results by the two methods (*P*>0.05). These results suggest the value of this TRFIA system for SSa detection in Chaihu crude drugs without any complicated pretreatments.

Although TRFIA has been used for at least 30 years, no reports have been available to describe its use in quantitative analysis of the bioactive constituents in traditional and folk Chinese crude drugs. We for the first time established a TRFIA system for SSa detection, which is the most sensitive method for such purposes so far. Given its high sensitivity, convenience and rapidness in high-throughput analysis, we believe that this technique has great potential in analysis of various Chaihu products and in detection of trace amount of SSa in different samples.

Previously, we developed several MAbs against bioactive constituents like ginsenosides [38], glycyrrizin in licorice [39], paclitaxel in yew trees [40], sennosides in rhubarb [41], and baicalin in *Scutellaria* [42]. These MAbs potentially allow the establishment of corresponding TRFIA systems for quality control and inspection of traditional Chinese drugs by detecting their bioactive components.

## Supporting Information

S1 File Raw data for curve fitting and SSa quantification. (XLS)
